# Spatial and temporal variation of epigaeic beetle assemblages (Coleoptera, Carabidae, Staphylinidae) in aspen-dominated mixedwood forests across north-central Alberta

**DOI:** 10.3897/zookeys.1044.65776

**Published:** 2021-06-16

**Authors:** H. E. James Hammond, Sergio García-Tejero, Greg R. Pohl, David W. Langor, John R. Spence

**Affiliations:** 1 Natural Resources Canada: Canadian Forest Service, Northern Forestry Centre, 5320 – 122 Street, Edmonton, AB, T6H 3S5, Canada Canadian Forest Service, Northern Forestry Centre Edmonton Canada; 2 Departamento de Biodiversidad y Gestión Ambiental, Universidad de León, Campus de Vegazana, 24195, León, Spain Universidad de Leon Leon Spain; 3 Department of Renewable Resources, University of Alberta, Edmonton, AB, T6G 2H1, Canada University of Alberta Edmonton Canada

**Keywords:** Climate, forest insect assemblages, insect biodiversity, spatial scale, variance partitioning, vegetation

## Abstract

Epigaeic beetle assemblages were surveyed using continuous pitfall trapping during the summers of 1992 and 1993 in six widely geographically distributed locations in Alberta’s aspen-mixedwood forests prior to initial forest harvest. Species composition and turnover (β-diversity) were evaluated on several spatial scales ranging from Natural Regions (distance between samples 120–420 km) to pitfall traps (40–60 m). A total of 19,885 ground beetles (Carabidae) representing 40 species and 12,669 rove beetles (non-AleocharinaeStaphylinidae) representing 78 species was collected. Beetle catch, species richness, and diversity differed significantly among the six locations, as did the identity of dominant species. Beetle species composition differed significantly between the Boreal Forest and Foothills Natural Regions for both taxa. Staphylinidae β-diversity differed significantly between Natural Regions, whereas Carabidae β-diversity differed among locations. Climate variables such as number of frost-free days, dry periods, and mean summer temperatures were identified as significant factors influencing beetle assemblages at coarse spatial scales, whereas over- and understory vegetation cover, litter depth, shade, slope, and stand age influenced beetle assemblages at finer spatial scales. Significant interannual variation in assemblage structure was noted for both taxa. Because composition of epigaeic beetle assemblages differed across spatial scales, forest management strategies based only on generalized understanding of a single location will be ineffective as conservation measures. In addition, site history and geographic variation significantly affect species distributions of these two beetle families across the landscape. Thus, we underscore Terry Erwin’s suggestion that biodiversity assessments focused on species assemblages at different spatial scales provide a sound approach for understanding biodiversity change and enhancing conservation of arthropod biodiversity.

## Introduction

Terry Erwin’s studies of the flora and fauna of tropical forests have profoundly influenced global efforts to understand forest biodiversity. In his classic paper that has inspired people to pursue questions about insect biodiversity Erwin (1982) clearly recognized the importance of β-diversity for estimating the number of species that should be expected in an area of forest. At this time, few data about β-diversity were available to Erwin and only approximate guesses about such relationships were possible. Erwin’s admirable efforts were mainly spent documenting and organizing information about species in the tropical forests that were his passion. He recognized successful conservation strategies and advocated approaches that were based on an understanding of β-diversity at different spatial scales (Erwin 1982, 1991a, b). Erwin’s later work would go on to show that β-diversity can change dramatically over relatively small spatial scales and that the fauna responds to large-and small-scale differences in macro- and microhabitat (Erwin et al. 2005). Of course, before one can understand faunal change, it is fundamentally necessary to know and be able to recognize the species involved. Now, 40 years later, for groups in which the α-taxonomy challenges have been largely met, it should be possible to quantify how faunas change over space and better predict how such changes are associated with environmental parameters subject to measurement.

Unlike the tropical forests that Terry studied, where overstory tree richness often exceeds 200 species per hectare (Pitman et al. 2001), the western Canadian boreal forest is dominated by only nine locally dominant tree species (Farrar 1995; Johnson et al. 1995). The mixedwood cover type, extending from Manitoba to northwestern British Columbia, is the dominant forest type in the province of Alberta > 290,000 km^2^ (Pratt and Urquhart 1994). Natural disturbances, especially wildfire, have played a historically important role in determining age structure, species composition, and succession in these forests (Johnson et al. 1998). On upland sites, which produce most of the region’s merchantable timber, these forests are dominated by a combination of *Populus* species, especially trembling aspen (*Populustremuloides* Michaux, 1803), and white spruce (*Piceaglauca* (Moench) Voss, 1907). There is much variability among sites with respect to the presence and abundance of other species such as balsam poplar (*Populusbalsamifera* Linnaeus, 1753), paper birch (*Betulapapyrifera* Michaux, 1803), black spruce (*Piceamariana* (Miller) Britton Sterns & Poggenburg, 1888), balsam fir (*Abiesbalsamea* (Linnaeus) Miller, 1768), lodgepole pine (PinuscontortaDougl. & Loudonvar.latifolia Engelmann, 1838), and jack pine (*Pinusbanksiana* Lambert, 1803). These species are generally minor canopy elements but may dominate some local sites depending on soil moisture, elevation, and aspect (Peterson and Peterson 1994; Beckingham and Archibald 1996; Beckingham et al. 1996). Thus, the boreal mixedwood has evolved as a mosaic of forest stands that vary in size, shape, age, soil moisture and nutrient regime, and vegetation characteristics. Industrial-scale forestry began to affect these stands in Alberta, mainly with respect to the spruce component, shortly after World War II; however, more recently large landscape-scale effects on the mixedwood forest system were initiated with construction of mills to exploit the *Populus* components of the forest in the mid to late 1980s (Pratt and Urquhart 1994).

The typical upland mixedwood succession pattern in western Canada is for deciduous species, mainly trembling aspen, to dominate upland stands as they recover from disturbance. Such aspen-dominated forests eventually give way to variable mixes of deciduous and coniferous trees and finally to conifer-dominated (usually white spruce) stands in the prolonged absence of wildfire and depending on availability of conifer seed sources (Rowe 1972; Stewart et al. 2001; Solarik et al. 2010). However, progression of this typical succession can be delayed for extended periods by local disturbance and associated within-stand processes such as gap dynamics so that *Populus* spp. remain the dominant trees in particular mixedwood stands for centuries (Cumming et al. 2000).

Boreal mixedwood stands provide extensive habitats for a wide range of forest biodiversity in western Canada. Although taxonomy, distribution, and habitat affinity of some groups such as plants and vertebrates are well understood, relatively little information is available for hyper-diverse groups such as invertebrates. In fact, only the small subset of species that cause economic damage to trees, biting insects, and a few charismatic groups such as butterflies and dragonflies are reasonably well known. Thus, the poor state of knowledge about most of the biodiversity in these forests is especially concerning as these landscapes are widely impacted by cumulative effects of timber harvest, oil and gas exploration, climate change, and changing fire cycles and intensity (Attiwill 1994; Haeussler and Kneeshaw 2003; Burton et al. 2006). The lack of knowledge concerning species composition, distribution patterns and ecological features of mixedwood communities challenges our ability to assess and model biodiversity changes in the wake of natural or anthropogenic disturbances. Present knowledge is simply insufficient to establish appropriate biodiversity goals for landscape restoration and reclamation, and predicting the long-term implications of large-scale industrial development of these forests will be difficult, especially against a backdrop of ongoing environmental change. The Canadian approach to modern sustainable forest management (CCFM 2003) requires viewing a variety of organisms through a broad lens of geographic and temporal scales to ensure biodiversity is conserved. Clearly, increasing information about biodiversity of functionally diverse groups such as invertebrates is both relevant and important to address conservation of biodiversity, despite the associated taxonomic challenges (Spence et al. 2008).

Amongst terrestrial invertebrates, epigaeic beetles, especially Carabidae, have been popular subjects for disturbance ecology research globally (Magura et al. 2003; Bus de Warnaffe and Lebrun 2004; Osawa et al. 2005; Work et al. 2008; Grove 2010; Lange et al. 2014; Baker et al. 2016). In North America, the earliest research about conservation of the two pre-dominant epigaeic beetle taxa in forests, Carabidae (ground beetles, Niemelä et al. 1993) and Staphylinidae (rove beetles, Pohl et al. 2007, 2008), was done in Alberta and has included work in the boreal mixedwood (e.g., Langor et al. 1994; Spence et al. 1996). Since then > 100 studies of these beetles have been conducted in the forests of Canada (Langor et al., in prep.), including many in the western boreal forest in a variety of cover-types.

One of the earliest studies, conducted in 1992–1993, addressed spatial variation in the structure of epigaeic beetle assemblages in aspen-dominated forests of Alberta, as extensive commercial harvesting of aspen had already begun. The most urgent conservation-oriented aspect of this work focused on whether old growth aspen-dominated forests (> 100 years post-fire) harbored significantly different assemblages than did mature forests (50–80 years) (Spence et al. 1996), as this was relevant to setting rotation ages for extensive harvests in Alberta. Another portion of this effort that sought to understand spatial variation in epigaeic beetle assemblages, i.e., β-diversity, in mature-to-old aspen stands across several Natural Regions and Subregions in north-central Alberta, remains unpublished. Although these data were collected almost 30 years ago, no more recent comparable studies exist and, as above, these earlier datasets provide baselines fundamental to evaluating post-disturbance forest recovery in the wake of forest management, oil and gas development and climate change. Publication of this baseline will support assessment of how anthropogenic and natural disturbances in Alberta’s Boreal and Cordilleran forests have affected species composition and β-diversity in these beetle groups.

In this paper, we examine composition and structure of carabid and staphylinid assemblages to determine (1) whether Natural Regions, Subregions, and stand age are useful predictors of arthropod assemblage structure, (2) β-diversity within Natural Regions, Subregions or stands of the same age at different spatial scales, (3) the main patterns of arthropod community composition at different spatial scales and the extent to which they are explained by environmental variables, and (4) how such information can best contribute to effective arthropod conservation.

## Materials and methods

### Study areas and forest features

**Figure 1.**
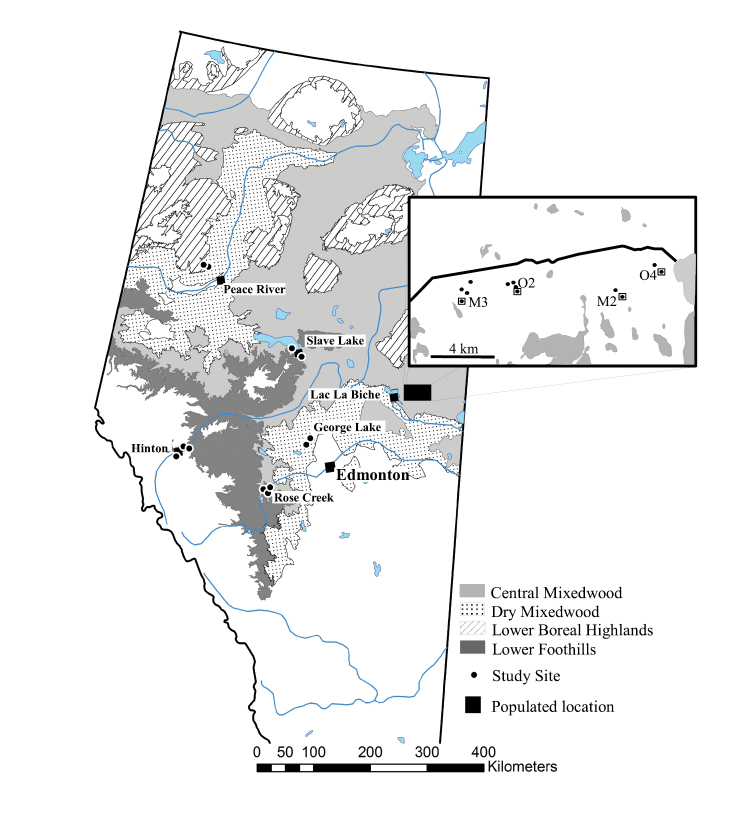
Location of aspen-dominated mixedwood study sites sampled in north-central Alberta, 1992 and 1993. INSET: location of individual pitfall trap lines at Lac la Biche, with lines used in the regional dataset surrounded with box.

Assemblages of Carabidae and Staphylinidae were sampled from the forest floor in sites from six locations in Alberta (Fig. 1, Table 1). We used Regions and Subregions as defined by the province (Downing and Pettapiece 2006) to classify our sites based on biophysical characteristics, mainly topography, climate, soils, and vegetation. Most of the *Populus*-dominated mixedwood sites fall into the Boreal Forest Natural Region, which is further subdivided into Natural Subregions. The George Lake sites lie within the Dry Mixedwood Subregion, the Peace River sites lie within the Lower Boreal Highlands Subregion, and the Rose Creek and Lac la Biche sites lie within the Central Mixedwood Subregion. The Hinton sites belong to the Lower Foothills Subregion of the Foothills Natural Region. Although the Slave Lake sites are located in the Central Mixedwood Subregion of the Boreal Forest Natural Region, they are very close to the border of the Lower Foothills Natural Region and thus represent a ‘transition’ zone between the two natural regions. We sampled two or three sites, separated by several kilometers in each Natural Region or Subregion (Table 1).

**Table 1.** Summary of forest stand characteristics of aspen mixedwood forests sampled by pitfall trap lines at six locations in north-central Alberta, 1992–1993.

Natural Region	Natural subregion	Location	Stands	General location	Stand age	Stand	Stand
				Latitude / Longitude	(years)	size (ha)	elevation (m)
Boreal Forest	Dry Mixedwood	George Lake	GLED	53.9564°N, -114.1233°W	80	60	693
Boreal Forest	Dry Mixedwood	George Lake	GLMC	53.9575°N, -114.1194°W	80	130	687
Foothills	Lower Foothills	Hinton	HIA	53.33308°N, -117.5977°W	80	5	1151
Foothills	Lower Foothills	Hinton	HIB	53.4864°N, -117.3825°W	75	100	1059
Foothills	Lower Foothills	Hinton	HIC	53.50452°N, -117.32148°W	85	20	1162
Boreal Forest	Central Mixedwood	Lac la Biche	M2 (2 lines)	54.8421°N, -111.4919°W	52	269	667
Boreal Forest	Central Mixedwood	Lac la Biche	M3 (4 lines)	54.8349°N, -111.657°W	51	315	642
Boreal Forest	Central Mixedwood	Lac la Biche	O2 (4 lines)	54.8458°N, -111.5873°W	125	134	649
Boreal Forest	Central Mixedwood	Lac la Biche	O4 (2 lines)	54.8531°N, -111.4473°W	122	187	691
Boreal Forest	Lower Boreal Highlands	Peace River	PRA	56.4131°N, -117.7308°W	80	38	731
Boreal Forest	Lower Boreal Highlands	Peace River	PRB	56.4042°N, -117.6856°W	70	37	710
Boreal Forest	Central Mixedwood	Rose Creek	RCA	53.05°N, -115.0578°W	105	10	819
Boreal Forest	Central Mixedwood	Rose Creek	RCB	53.0433°N, -115.1361°W	100	25	860
Boreal Forest	Central Mixedwood	Rose Creek	RCC	53.0428°N, -115.0714°W	95	10	835
Boreal Forest	Central Mixedwood	Slave Lake	SLA	55.2411°N, -114.7036°W	70	25	604
Boreal Forest	Central Mixedwood	Slave Lake	SLB	55.3372°N, -114.9567°W	125	20	603

We sampled mesic forest stands that were primarily dominated by *Populus* species, but which also contained mixes of scattered white spruce, paper birch, lodgepole pine, and willow (Suppl. material 1: Table S2). Coniferous trees generally represented < 5% of total stems in most stands, but accounted for 13% of trees in one stand near Hinton (HI-B). All stands had originated from natural wildfire and were at least 51 years old (Table 1), as determined by increment cores of the largest trees found near each trap line. Stands were generally 10 ha or larger with one exception (HI-A; Table 1).

### Beetle sampling

Epigaeic Carabidae and Staphylinidae were sampled using lines of six pitfall traps (Spence and Niemelä 1994) run continuously from 20 May to 14 October 1992 and 6 May to 20 October 1993. These periods encompassed nearly the entire frost-free season in both years, supporting confidence about composition of the local fauna and activity-based estimates of relative abundance of particular species across years (Baars 1979). Pitfall traps consisted of two nested white plastic containers 10 cm in diameter. The 1 L outer container served as a non-removable ‘sleeve’ dug into the ground with the top lip flush with the ground surface; this sleeve was permanently placed for the trapping season to minimize disturbance at the trap edge (Digweed et al. 1995; Bergeron et al. 2013). The 500 mL inner cup contained approximately 100 ml of ethylene glycol to serve as an insect killing agent and preservative, and it was removed to service the trap and collect the samples. Traps were covered with a 15 cm × 15 cm elevated plywood roof to exclude most precipitation and debris. Traps were visited at 10–20 day intervals throughout each season to remove accumulated insects, maintain the trap edge if necessary and replenish the preservative.

Most stands were sampled with one linear transect or ‘line’ of six traps. Within a line, traps were separated by ~ 50 m, which is sufficient to ensure independence of catch (Digweed et al. 1995). Trap lines were generally situated in the middle of stands and no traps were within 50 m of the stand edge. In the four large stands at Lac la Biche (M2, M3, O2, and O4; Fig. 1), either two or four lines were used to better sample variation within these stands for the comparison of stand-age effects. Because the trap lines in each stand were at least 300 m apart, we treated lines within stands at Lac la Biche as separate replicates in the analyses of β-diversity reported below.

All adult carabids and staphylinids were identified to species using taxonomic literature (primarily Lindroth 1961–69 for carabids; primarily Campbell 1969, 1973a, b, 1979, 1980, 1982a, b, 1983, 1984, 1991; Cuccodoro and Löbl 1996; Herman 1975; Smetana 1971, 1981, 1982, 1995 for staphylinids) and reference collections. Members of the staphylinid subfamily Aleocharinae were not identified nor considered in analyses because taxonomic resources are presently insufficient for reliable ecological work. Nomenclature follows Bousquet et al. (2013). Some genera of staphylinids included in our study need revision, and in these few cases we identified individuals to morpho-species within a genus. Voucher specimens are deposited in the Canadian Forest Service arthropod collection at the Northern Forestry Centre, Edmonton.

### Site characteristics

During the summer of 1992 we measured several site variables at trapping sites in each stand in order to test whether such features were correlated with beetle catches. Overstory and understory plant communities were assessed around each pitfall trap using a 5 m × 5 m vegetation plot centered on the trap. Tree cover was estimated as the number of stems > 5 m in height whose driplines fell into the 5 m × 5 m vegetation plot. Shrub density was measured by counting the shrub stems occurring at 0.5 m off the ground, in two 1 m wide transects, 5 m in length, one running north to south and the other running east to west, with the trap at the midpoints of both transects. Litter biomass (dried) was determined from a representative 0.25 m^2^ area chosen with the vegetation plot. We calculated other site characteristics such as southern aspect and slope using digital elevation models (DEM) downloaded from the Federal Geospatial Platform of the Government of Canada (url: https://ftp.maps.canada.ca/pub/nrcan_rncan/vector/index/html/geospatial_product_index_en.html#link). We calculated the southern aspect of the terrain around each trap as a proxy for solar irradiation, measuring how many degrees the orientation deviated from north, going from 0 (north) to 180 (south). Two different variables were computed: one considering only slopes > 2° and the other > 5°; terrain with slope < 2° or < 5° were considered as facing south and given a value of 180. Climatic data were downloaded from http://albertaclimaterecords.com/, which summarizes 30-year climatic averages from 1981 to 2010 on a 10 ×10 km provincial grid. These variables are summarized in Suppl. material 1: Tables S2, S3.

### Data analysis

Our entire dataset contains a total of 24 pitfall trap lines; however, because we sought to study several levels of variation in beetle assemblages, we separated the data into two subsets. The regional dataset contains all lines at all locations, except for Lac la Biche, where instead only four of the lines (two mature and two old from the 12 lines, Fig. 1, Table 1) were chosen. Thus, the regional dataset consists of 16 lines located in separate stands. The four lines at Lac la Biche were chosen because they made the lowest local contributions to β-diversity (LCBD), and therefore are closer to the stand centroid of values. The local dataset focused on the 12 pitfall trap lines from the four stands at Lac la Biche. We used the regional dataset to determine if Natural Regions and Subregions provide expectations about beetle assemblages across large spatial scales, and the local dataset to quantify the effect of stand age and fine spatial scale in shaping regional beetle assemblages.

Sampling effort varied among locations (range 62–176 days), due to different setup dates and losses attributable to trap disturbance by wildlife. Thus, the catch of each beetle species was standardized to 175 trap days [(raw abundance * (175 / actual number of trap days)]). These standardized abundance data were then subjected to the ‘Hellinger’ transformation, which reduces the ‘double zero problem’ of resemblance among samples, before preparing the data for linear analyses based on Euclidean distances (Legendre and Gallagher 2001; Borcard et al. 2018). Except for the temporal analysis, all subsequent analyses were performed on beetle catches averaged across years of collection to give a reasonably complete picture of the fauna at each region.

Differences in standardized abundance between years, ecoregions and locations were examined using a generalized linear mixed model (function glmer in R). Each trap catch was classified into a year, ecoregion and location, and formal hypothesis testing was done using pitfall trap line as a random effect. Effects were tested using Poisson, quasi-Poisson, and negative binomial distributions, and for each taxon the results were congruent for all error distributions based on Analysis of Deviance using Type II Wald Chi-square tests. In this paper we report the results based on the negative binomial model. Multiple comparisons tests were conducted using Tukey’s contrasts with a Holm adjustment to control experiment-wide error rate.

Beetle species richness among locations was compared using coverage-based rarefaction (Chao and Jost 2012) on Hill’s numbers q = 0 (species richness) and q = 1 (exponential of Shannon diversity). This method differs from the traditional sample-based rarefaction as it uses minimum sample completeness (coverage) rather than the lowest number of individuals collected in a sample as the threshold for comparison. Coverage equates to the percentage of the total number of individuals that belong to the observed species at the level of comparison desired. Species richness and diversity estimates were calculated at a level of > 97% completeness after 100 bootstrap randomizations extrapolated at twice the observed richness. We considered samples to differ significantly in species richness if the 95% confidence intervals of estimates did not overlap.

Spatial and temporal patterns of assemblages were examined using Principal Components Analysis (PCA). PCA reduces the dimensionality of multivariate data by constructing principal components, which are new variables that are uncorrelated, linear combinations of the original data. Principal components explain the maximum amount of variance in our community data in the fewest dimensions. In addition, interannual variation in the carabid and staphylinid assemblages was examined using pairwise Bray-Curtis measures of dissimilarity, transformed to percent similarity (1 – dissimilarity), on the standardized catch of the entire fauna, using species with a catch of greater than five individuals, and to presence-absence data for all species.

Redundancy Analysis (RDA) was used to ascertain if Natural Regions and Subregions (regional dataset) and stand age (local dataset) can be useful tools to classify the carabid and the staphylinid assemblages. The results were tested using 4999 permutations of the residuals, which were restricted to account for the nested model and fine-scale spatial autocorrelation (i.e., traps within lines were permuted along series in order to keep their spatial arrangement). When these classifications proved useful, we looked for significant indicator species for each group following the approach in De Cáceres et al. (2010). We used group-equalized indicator values to account for differences in sampling size between Natural Regions and Subregions. P-values were corrected for multiple comparisons using the Holm method. Significant indicator species (indicator value of at least 0.5 at α = 0.05) were identified after 4999 permutations.

Multivariate dispersion within each Region, Subregion, location, and line, and within each stand and each line for mature and old stands at Lac la Biche, was used to examine within-group β-diversity. We then tested for differences between Natural Regions, Subregions or stands of different age using restricted permutations (see above).

To explore the main patterns in the data we used multivariate regression trees (De’ath 2002), with line identity as explanatory variable. This allowed us to freely model variation between lines, leaving variation within lines untouched, and provided simpler and clearer dendrograms than alternative unconstrained classification methods. Since line identity is a meaningless variable cross-validation could not be used as a proper method to prune the tree. Instead, we coded the branches at each node of the tree as dummy variables and used RDA to test for significance of each split, leaving only the nodes that were significant or marginally significant. This resulted in informative but small and parsimonious trees.

Variables about climate and habitat structure were used to explain patterns at the Natural Region scale. We first forward selected the climatic variables in RDA according to the amount of variation that each could explain and tested the results using restricted permutations (see above). Only the first two variables were kept in the model to avoid overfitting, and since our sites are clustered, there were only eleven different values for each climatic variable. Keeping these two variables in the model, we included habitat structure variables by forward-selection. Tests in this second case were done without restricting the permutations since environmental variables were measured at each trap and we expect that they may be the cause of possible spatial autocorrelation patterns in the beetle data (i.e., we assume the “environmental control” model, see Declerck et al. 2011). For the Lac la Biche dataset we used the same approach, but only with habitat structure variables.

Variation partitioning was used to combine results from environmental RDA models, together with dummy variables coding for locations and lines in the regional dataset, and for stands and lines, in the Lac la Biche dataset. This allowed us to better understand how the main patterns of variation are arranged across spatial scales and the extent to which they can be explained by environmental variables.

All beetle species and community analysis were computed with RStudio running R version 4.0.2 (R Core Team 2020) using the following packages: ade4 v1.7-16 (Dray et al. 2017); adegraphics v1.0-15 (Siberchicot et al. 2017); adespatial v0.3-8 (Dray et al. 2020); ape v5.4-1 (Paradis and Schliep 2019); iNEXT v2.0.20 (Hsieh et al. 2020); indicspecies v1.7.9 (De Cáceres and Legendre 2009); mvpart v1.6-2 (Therneau et al. 2014); SoDA v1.0-6.1 (Chambers 2020); and vegan v2.5-6 (Oksanen et al. 2019).

## Results

### Catch, species diversity, and beetle assemblage patterns

**Figure 2.**
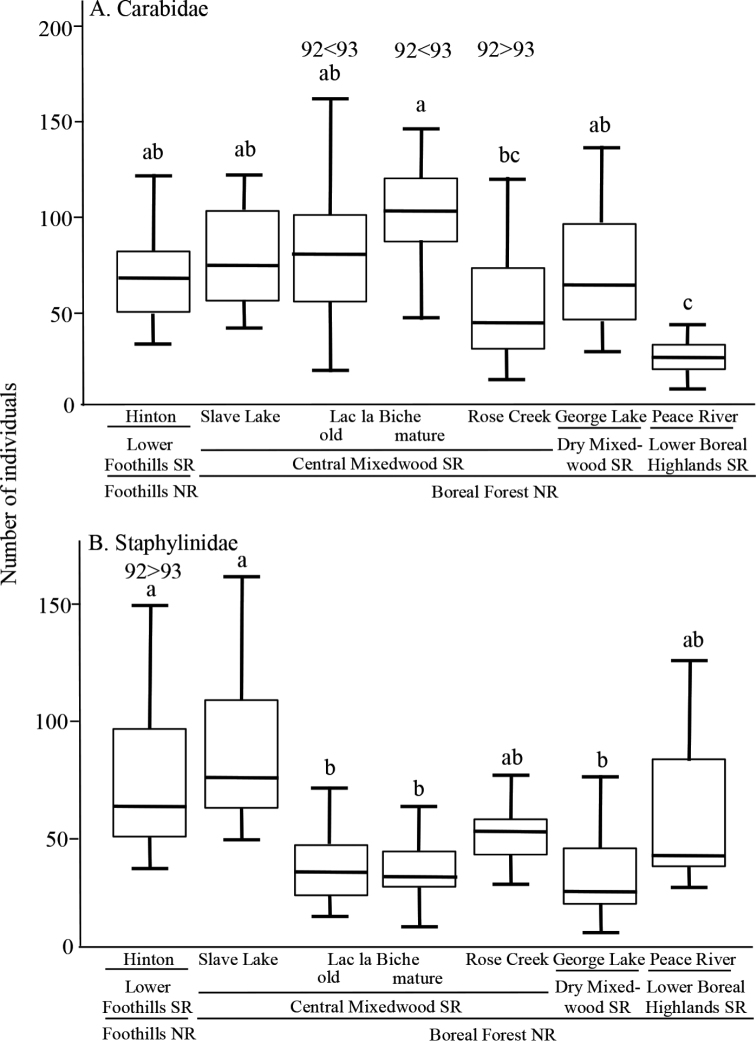
Boxplot of the standardized catch of epigaeic beetles at seven locations in aspen-dominated mixedwood forests in north-central Alberta, 1992–93. Locations are distributed across four Natural Subregions (SR) and two Natural Regions (NR) **A**Carabidae**B**Staphylinidae. Locations with the same letter situated above the bar are not significantly different at α = 0.05 (post hoc Tukey’s tests). For locations where there was significant interannual variation, the results are indicated above the bar for that location.

A total of 32,554 epigaeic beetles was collected during the summers of 1992 and 1993, of which 61% were Carabidae and 39% Staphylinidae (excluding Aleocharinae; Suppl. material 1: Table S1). Mean standardized catches of Carabidae (78.2 ± 2.81 per 175 trap days) were significantly greater (χ^2^ = 74.7, p < 0.001) than for Staphylinidae (51.3 ± 2.02). Mean trap catches of both taxa varied significantly across Subregions (Carabidae: χ^2^ = 12.5, p = 0.006; Staphylinidae: χ^2^ = 7.6, p = 0.050) and locations (Carabidae: χ^2^ = 38.7, p < 0.001; Staphylinidae: χ^2^ = 49.5, p < 0.001). Carabid catch was lowest at Peace River in the Lower Boreal Highlands Subregion, but relatively similar among the other ecoregions and locations (Fig. 2A); whereas catches of staphylinids were significantly lower at George Lake in the Dry Mixedwood Subregion and the Lac la Biche stands of the Central Mixedwood Subregion, and highest at Hinton and Slave Lake locations (Fig. 2B).

Of the 40 species of Carabidae and 78 species of Staphylinidae captured across all locations and years, all but three species are native. The non-native Palearctic carabid, *Pterostichusmelanarius* (Illiger, 1798), was collected at two locations in this study: George Lake (both stands; ca. 5% of total catch) and the HIB stand at Hinton (one specimen). The George Lake stands are embedded in an agricultural setting and *P.melanarius* was first documented as an invader of these stands in 1981 (Niemelä and Spence 1991). The HIB stand was also located close to human habitation and pastures. Other sampled stands are remote from anthropogenic habitats so there was little opportunity for invasion by non-native species. Two additional species, *Philonthusconcinnus* (Gravenhorst, 1802) and *P.cruentatus* (Gmelin, 1790), are common non-native species associated with disturbed ground and are often collected in cattle and horse dung.

**Figure 3.**
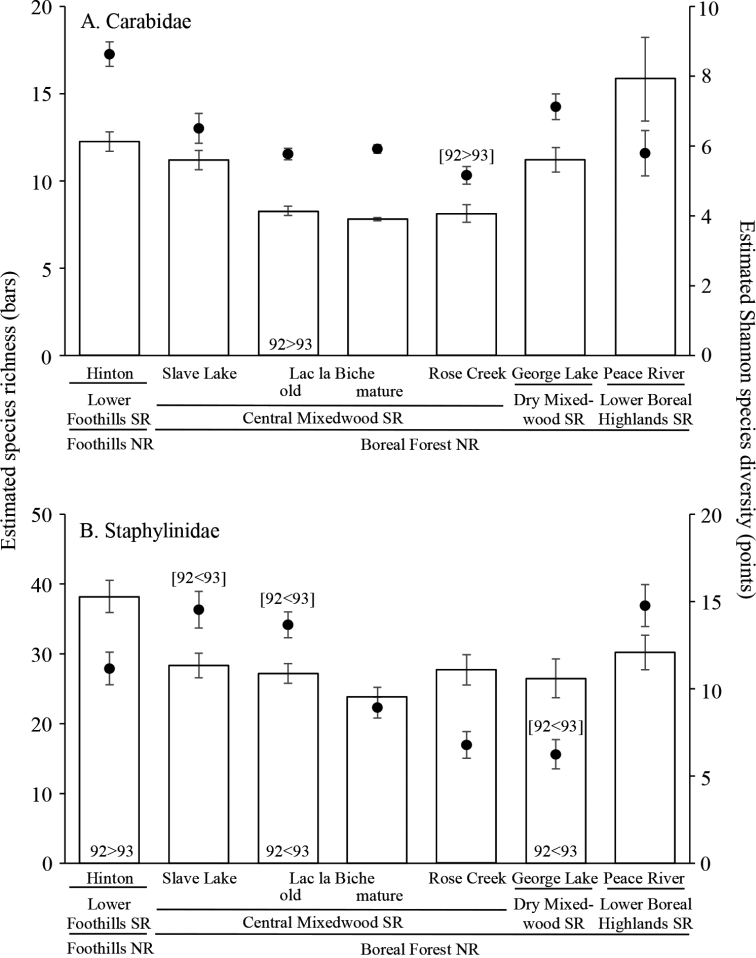
Coverage based rarefaction estimates of species richness and diversity of epigaeic beetles at each location in aspen-dominated mixedwood forests of north-central Alberta, 1992–93. Locations are distributed across four Natural Subregions (SR) and two Natural Regions (NR) **A**Carabidae (97.2% coverage) **B**Staphylinidae (97.6% coverage). Bars represent species richness (± 95% CI) and points represent the exponential of the Shannon diversity index (± 95% CI). For locations where there was significant interannual variation, the results for species richness are placed at the bottom of each bar, and results for diversity are placed in square brackets [] and are indicated above the point for that location.

Rarefaction-estimated species richness of Carabidae was highest at Peace River in the Lower Boreal Highlands Subregion, despite the fact that catch was lowest there (Fig. 3A). However, carabid species diversity, as measured with the Shannon diversity index, was highest in the Lower Foothills Subregion (Hinton location) and lowest at Rose Creek in the Central Mixedwood Subregion, although the differences between the four locations in the Central Mixedwood Subregion were relatively small (Fig. 3A). Rarified staphylinid species richness was highest in the Lower Foothills Subregion (Hinton); however, Shannon diversity was highest in the more northern locations, Slave Lake, Lac la Biche, and Peace River, and lowest at the more southern Rose Creek and George Lake locations (Fig. 3B).

The dominant Carabidae included: *Pterostichusadstrictus* Eschscholtz, 1823 (23.0% of overall catch), *Pterostichuspensylvanicus* LeConte, 1873 (20.8%), *Calathusingratus* Dejean, 1828 (11.4%), and *Platynusdecentis* (Say, 1823) (9.3%) comprising > 5.0% of total catch at every location. *Agonumretractum* LeConte, 1846 (13.9%) and *Scaphinotusmarginatus* (Fischer von Waldheim, 1820) (8.2%) were abundant at four of six locations (Suppl. material 1: Table S1). The dominant species of Staphylinidae included: *Tachinusfumipennis* (Say, 1832) (23.6%), *Staphylinuspleuralis* LeConte (13.0%), *Tachinusfrigidus* Erichson, 1839 (7.7%), and *Quediusu.uteanus* (Casey, 1915) (7.7%), but none of them represented > 5.0% of catch at all seven locations. *Tachinusfrigidus* and *T.elongatus* Gyllenhal, 1810, for example, were abundant only at Hinton, where they largely replaced *T.fumipennis*, which was abundant at all other locations. *Habrocerusschwarzi* Horn, 1877 (5.9%) and *Quediusrusticus* Smetana, 1971 (5.5%) were abundant at four of seven locations (Suppl. material 1: Table S1). Six additional species of Carabidae and five species of Staphylinidae were abundant at different locations, indicating that the dominance structure of epigaeic beetle assemblages varied considerably, likely reflecting local environmental or microhabitat conditions.

Many species were uncommonly collected over the course of the study; eleven species of Carabidae and 20 species of Staphylinidae were singletons or doubletons in our data set, and an additional eleven species of Carabidae and 23 of Staphylinidae were represented by < 15 specimens (Suppl. material 1: Table S1). Among Carabidae represented by only one or two specimens, most appeared to be incidental captures of species that are not typically found in mature mesic forests, including three hygrophilic species (*Agonumconsimile* (Gyllenhal, 1810), *Agonumpropinquum* (Gemminger & Harold, 1868), and *Elaphrusclairvillei* Kirby, 1837) and five species characteristic of open habitats (four *Amara* species and *Harpaluslaevipes* Zetterstedt, 1828). In general, habitat associations of the uncommonly collected Staphylinidae are poorly known, but among those for which there is some information, seven species are thought to be hygrophilic: *Olophrumrotundicolle* (C.R. Sahlberg, 1830), *Tachyporusrulomus* Blackwelder, 1936, *Ischnosomapictum* (Horn, 1877), *Stenusimmarginatus* Mäklin, 1853, *Stenusrossi* Sanderson, 1958, *Stenussibiricus* J. Sahlberg, 1880, and *Gabriuspicipennis* (Mäklin, 1852). *Quediusplagiatus* Mannerheim, 1843 and *Atrecusmacrocephalus* (Nordmann, 1837) are thought to live under the bark of dead or dying trees, and so while they may be common microhabitat specialists in boreal forest, they are not typically collected in pitfall traps deployed on the ground. *Bisniuscephalicus* (Casey, 1915) has been globally known from only one specimen until recently (Smetana 1995); however, it has since been collected at several locations in the boreal forest of western Canada. One species each of *Bolitobius* (136 specimens) and *Arpedium* (one specimen) collected in the current study appear to represent undescribed species.

### Regional scale assemblage structure and β-diversity

**Figure 4.**
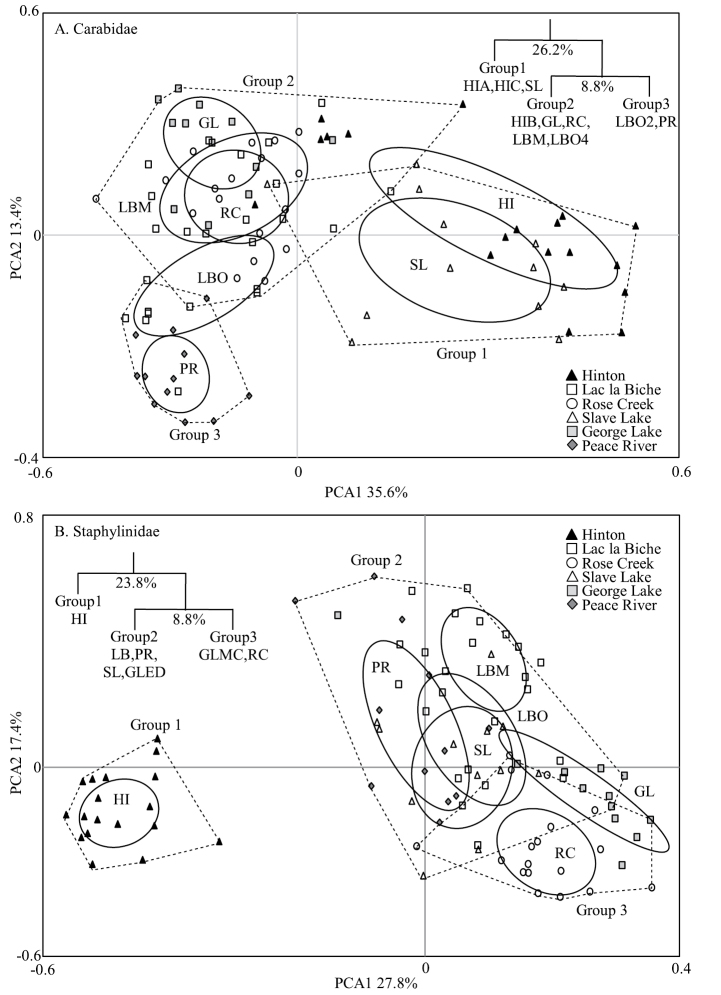
Principal components analysis of epigaeic beetle assemblages in aspen-dominated mixedwood forests in north-central Alberta, 1992–93 **A**Carabidae**B**Staphylinidae. Each symbol represents the catch from a single pitfall trap averaged over two years. Black symbols represent the Lower Foothills Natural Region; open symbols represent the Central Mixedwood Subregion, light grey symbols represent the Dry Mixedwood Subregion and the dark grey symbols represent the Lower Boreal Highlands Subregion of the Boreal Forest Natural Region. Ellipses represent the standard deviation around the centroid of points in each group and dashed polygons represent the final nodes of the multivariate regression tree (MRT) model. The inset shows the major MRT nodes grouping lines based on assemblage structure.

Natural Region significantly explained assemblage structure for carabids (F_1,94_ = 21.06, p = 0.006) and staphylinids (F_1,94_ = 29.42, p = 0.001), accounting for 17.4% and 23.0%, respectively, of variation in the data (Fig. 4). Carabid assemblages also significantly differed between Subregions of the Boreal Forest Natural Region (F_2,92_ = 9.69, p = 0.030), while staphylinids did not (F_2,92_ = 5.14, p = 0.097) (Fig. 4). Carabid assemblages of the Lower Boreal Highlands Subregion (Peace River) significantly differed from those of the remaining Boreal Forest Subregions (F_1,76_ = 10.23, p = 0.039), and these differences accounted for 6.9% of the total variation in the carabid data at the regional scale (Fig. 4A). The first two axes of the PCA accounted for 49.0% of the variability in the observed variation in Carabidae and 45.2% of that for the Staphylinidae (Fig. 4A, B).

Multivariate regression tree analysis (MRT) of the carabid data by lines resulted in a tree with two significant splits (split 1: F_1,82_ = 71.2, p < 0.001; split 2: F_1,82_ = 25.7, p = 0.033) forming three clusters: group 1, which included the assemblages from the Hinton HIA and HIC lines and those from Slave Lake; group 2, which included the Hinton HIB line, and those from George Lake, Rose Creek, Lac la Biche Mature, and the O4 line at Lac la Biche; and group 3, which included the Peace River lines and the O2 line at Lac la Biche (Fig. 4A, inset). The nodes accounted for 26.2% and 8.8%, respectively, of the among-line variation in carabid assemblages. The MRT of the Staphylinidae data also resulted in a tree with two significant splits (split 1: F_1,85_ = 43.6, p < 0.001; split 2: F_1,85_ = 17.8, p = 0.007) forming three clusters: group 1, which included all Hinton lines; group 2, which included all lines from Lac la Biche, Peace River, Slave Lake, and the George Lake ED line; and group 3, which included all Rose Creek lines and the George Lake MC line (Fig. 4B, inset). The nodes accounted for 23.8% and 8.8% of the among-line variation in staphylinid assemblages. Therefore, the two beetle families had somewhat different patterns of assemblage variability among and within locations across the landscape suggesting that carabid and staphylinid assemblages respond differently to environmental parameters characterizing these local forest ecosystems.

Eleven carabid and 17 staphylinid species were identified as significant indicator species (Table 2); carabid species were associated with the Lower Foothills Natural Region (5 species), or the Dry Mixedwood Subregion (4 species), whereas staphylinids were indicators of the Lower Foothills Natural Region (6 species), the Boreal Forest Natural Region (3 species) and the Lower Boreal Highlands Subregion (4 species), or combinations of Regions or Subregions (4 species). The carabid and staphylinid species with the highest indicator values (IV) were, respectively, *A.retractum* (IV = 0.975) and *T.fumipennis* (0.970), and both were significant indicators of the Boreal Forest Natural Region (Table 2).

**Table 2.** Carabidae and Staphylinidae species identified as indicators of a particular Natural Region, Subregion, or combination.

Family	Carabid species	Natural Region (NR), Natural Subregion (SR), or combination	Indicator value*	p-value**
Carabidae	*Calosomafrigidum* Kirby, 1837	Foothills NR	0.826	0.007
	*Calathusadvena* (LeConte, 1846)	Foothills NR	0.647	0.007
	*Leistusferruginosus* Mannerheim, 1843	Foothills NR	0.638	0.007
	*Pterostichusriparius* (Dejean, 1828)	Foothills NR	0.822	0.007
	*Trechuschalybeus* Dejean, 1831	Foothills NR	0.776	0.007
	*Agonumretractum* LeConte, 1846	Boreal Forest NR	0.975	0.007
	*Agonumcupreum* Dejean, 1831	Dry Mixedwood SR	0.490	0.043
	*Harpalusfulvilabris* Mannerheim, 1853	Dry Mixedwood SR	0.861	0.007
	*Pterostichusmelanarius* (Illiger, 1798)	Dry Mixedwood SR	0.813	0.007
	*Synuchusimpunctatus* (Say, 1823)	Dry Mixedwood SR	0.951	0.007
	*Scaphinotusmarginatus* (F.v. Waldheim, 1820)	Central Mixedwood SR + Dry Mixedwood SR + Foothills NR	0.919	0.007
Staphylinidae	*Micropepluslaticollis* Mäklin, 1853	Foothills NR	0.527	0.034
	*Philonthusvarians* (Paykull, 1789)	Foothills NR	0.615	0.024
	*Quediusm.molochinoides* Smetana, 1965	Foothills NR	0.737	0.014
	*Tachinusfrigidus* Erichson, 1839	Foothills NR	0.948	0.014
	*Tachinusquebecensis* Robert, 1946	Foothills NR	0.770	0.014
	*Tachyporusabdominalis* (Fabricius, 1781)	Foothills NR	0.577	0.034
	*Lathrobiumfauveli* Duvivier, 1883	Boreal Forest NR	0.768	0.034
	*Quediusl.labradorensis* Smetana, 1965	Boreal Forest NR	0.930	0.014
	*Tachinusfumipennis* (Say, 1832)	Boreal Forest NR	0.970	0.014
	*Ischnosomasplendidum* (Gravenhorst, 1806)	Lower Boreal Highlands SR	0.893	0.014
	*Mycetoporusamericanus* Erichson, 1839	Lower Boreal Highlands SR	0.819	0.014
	*Philonthusflavibasis* Casey, 1915	Lower Boreal Highlands SR	0.554	0.043
	*Quediusbrunnipennis* Mannerheim, 1843	Lower Boreal Highlands SR	0.615	0.024
	*Habrocerusschwarzi* Horn, 1877	Dry Mixedwood SR + Lower Boreal Highlands SR	0.836	0.043
	*Quediusfulvicollis* (Stephens, 1833)	Foothills NR + Lower Boreal Highlands SR	0.766	0.043
	*Quediusvelox* Smetana, 1971	Foothills NR + Lower Boreal Highlands SR	0.860	0.014
	*Tachinuselongatus* Gyllenhal, 1810	Foothills NR + Lower Boreal Highlands SR	0.919	0.014

* We used group-equalized indicator values to account for differences in sampling effort between groups. **P-values were corrected for multiple comparisons using the Holm method.

**Figure 5.**
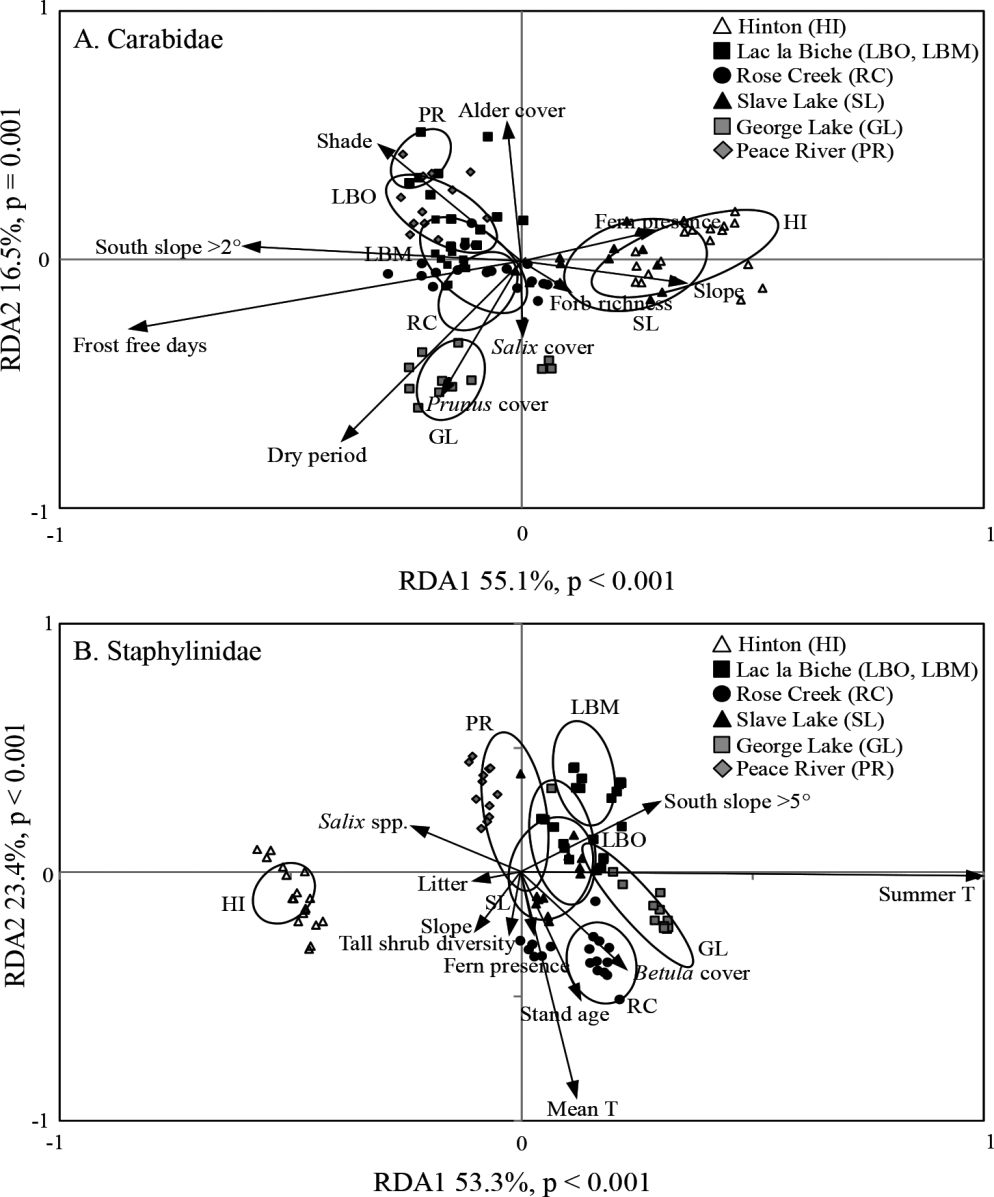
Redundancy analysis of epigaeic beetle assemblages in aspen-dominated mixedwood forests in Alberta, 1992–93 **A**Carabidae**B**Staphylinidae. Each symbol represents the catch from a single pitfall trap averaged over two years. Open symbols represent the Foothills Natural Region; the black symbols represent the Central Mixedwood Subregion, grey squares represent the Dry Mixedwood Subregion and the grey diamonds represent the Lower Boreal Highlands Subregion of the Boreal Forest Natural Region. Ellipses represent the standard deviation around the centroid of points in each group.

For carabids, the two climatic variables included in the environmental model, the number of frost-free days and the length of the normal dry period, together accounted for 27.7% of the total variation (Fig. 5A). Subsequently selected habitat structure variables, southern aspect > 2°, total shade, presence of ferns, *Salix* spp. cover, slope, *Alnus* and *Prunus* spp. cover, and forb diversity, together accounted for 44.7% of total variation, with axes 1 and 2 explaining 55.1% and 16.5% of the variance, respectively (Fig. 5A). Carabid assemblages from boreal locations (excluding Slave Lake) were positively correlated with the number of frost-free days and south facing slopes > 2°, whereas those from Hinton and Slave Lake were positively correlated with amount of forb and fern cover.

For staphylinids, the two climatic variables included in the environmental model were mean summer temperature and mean annual temperature, which together accounted for 32.0% of the total variance (Fig. 5B). Subsequently selected habitat structure variables – stand age, *Salix* spp. cover, slope, presence of ferns, diversity of tall shrubs, birch cover, and litter biomass – together accounted for 40.9% of total variation, with axis 1 and 2 explaining 53.3% and 23.4% of the variance, respectively (Fig. 5B). Staphylinidae from the boreal locations were strongly correlated with mean summer temperature, and to a lesser extent south facing slopes > 5° which is a characteristic feature of the mound and kettle geography of eastern Alberta. The analysis identified no strong correlations between environmental and climate variables for staphylinids in the lower foothills.

**Figure 6.**
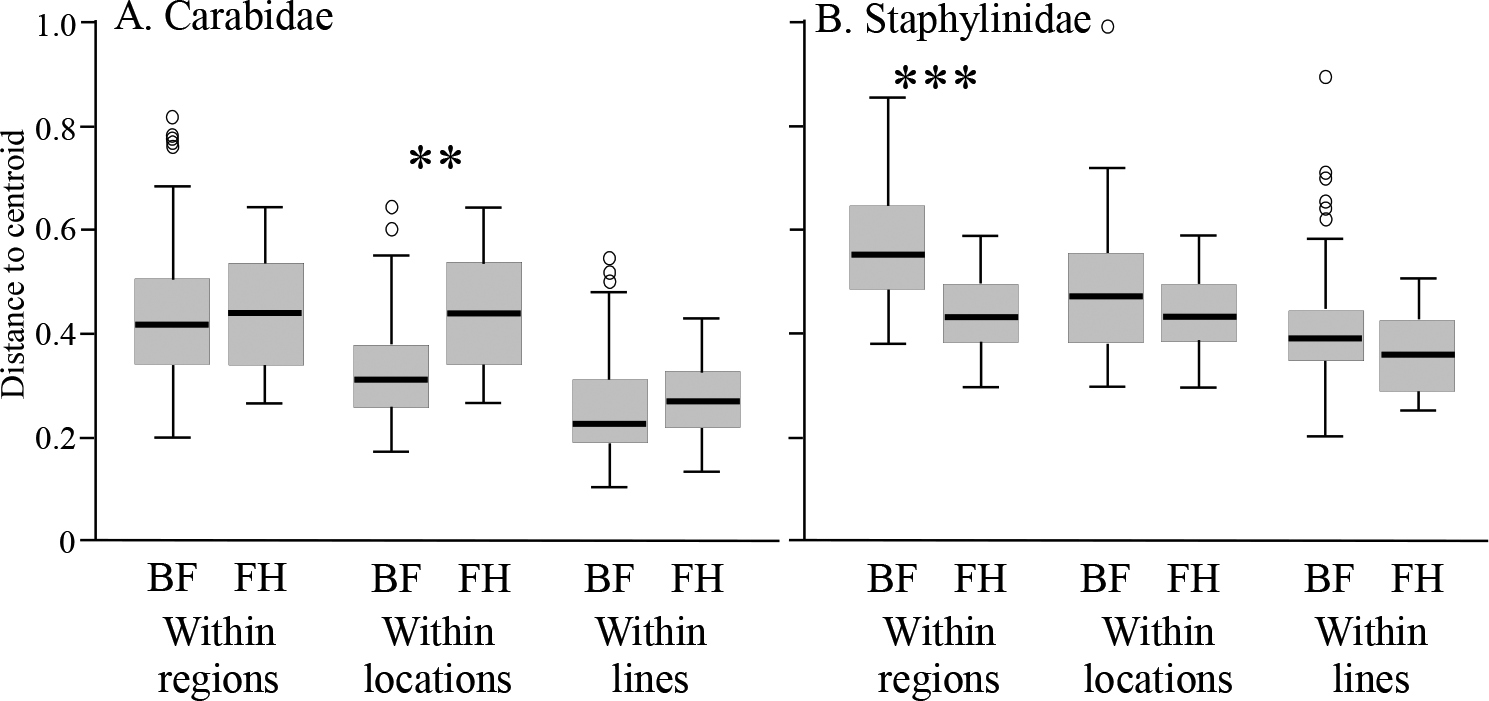
Boxplots of epigaeic beetle assemblage beta-diversity within regions, within locations and within lines for Boreal Forest (BF) and Foothills (FH) Natural Regions **A**Carabidae**B**Staphylinidae. Significant differences between Natural Regions are indicated by asterisks above the boxplots (** p < 0.01, *** p < 0.001).

Overall β-diversity, including variation between locations, did not differ significantly between Natural Regions for carabids (F_1,94_ = 0.08, p = 0.893; Fig. 6A) but it was significantly higher in the Boreal Forest Natural Region than in the Lower Foothills Subregion for staphylinids (F_1,94_ = 23.21, p < 0.001; Fig. 6B). However, carabids showed significantly higher β-diversity at Hinton in the Lower Foothills Subregion than in locations in the Boreal Forest Natural Region (F_1,94_ = 21.44, p = 0.007; Fig. 6A), although there was no similar significant difference for staphylinids (F_1,94_ = 1.19, p = 0.411; Fig. 6B). β-diversity within lines did not significantly differ between Natural Regions for either carabids (F_1,94_ = 1.08, p = 0.348; Fig. 6A) or staphylinids (F_1,94_ = 2.62, p = 0.137; Fig. 6B). Subregions within the Boreal Forest Natural Region showed no significant differences in β-diversity at any scale.

**Figure 7.**
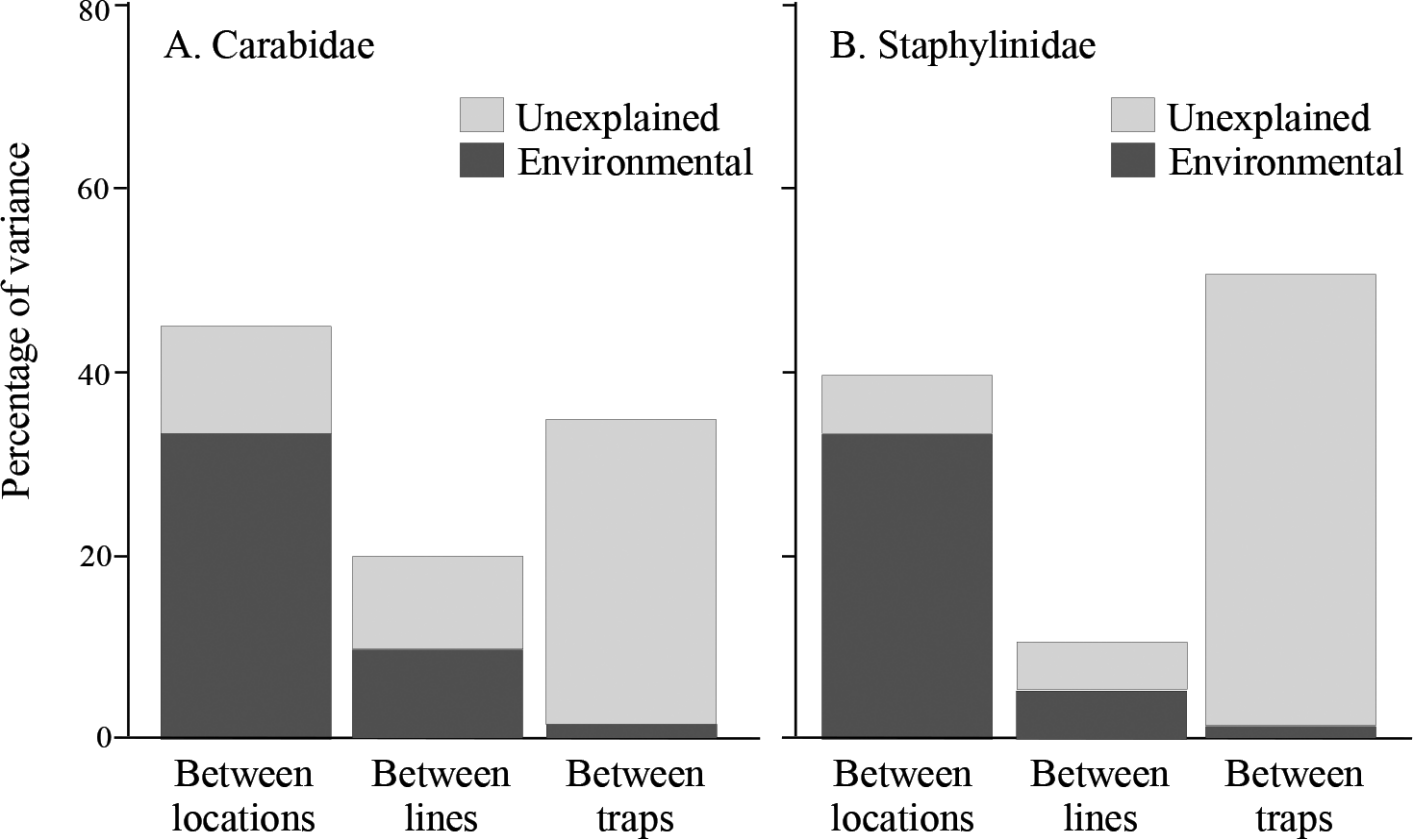
Adjusted percentage of variance of assemblage composition in the regional dataset explained at each spatial scale – between locations, between lines in the same location and between traps in the same line **A**Carabidae**B**Staphylinidae.

Total variance was lowest between lines for both beetle groups (carabids: 20.1%; staphylinids: 9.9%; Fig. 7), but the highest source of variance differed between the two beetle families. For carabids, variance between locations was highest at 45.1% and for staphylinids between-trap variance was highest at 50.4%. However, environmental parameters explained the highest proportion of variance between locations for both taxa (carabids: 74.3%; staphylinids: 83.9%), followed by variance explained between-lines and with the lowest proportion of variance explained between-traps.

### Local scale assemblage structure and β-diversity at Lac la Biche

**Figure 8.**
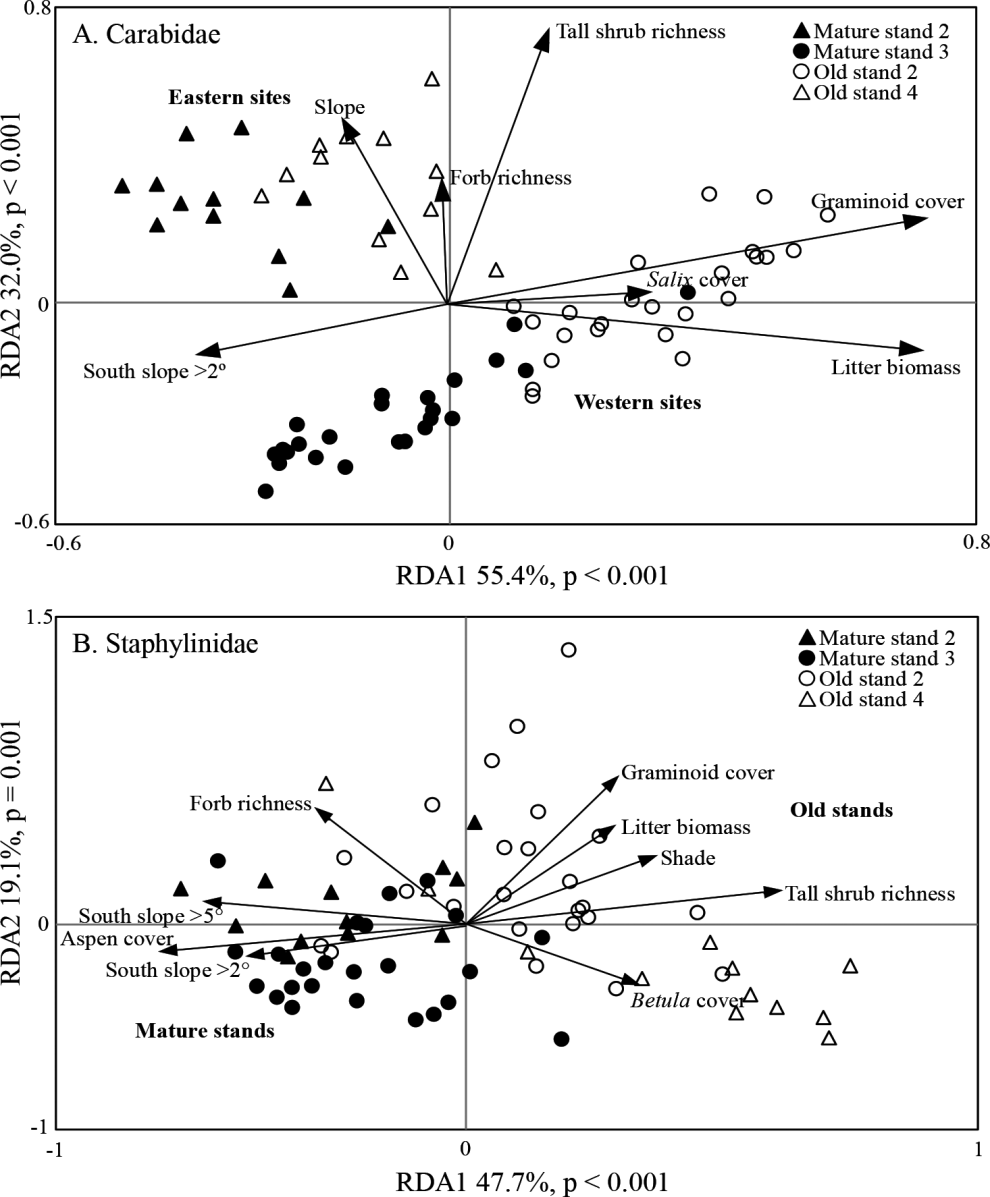
Redundancy analysis (RDA) of epigaeic beetle assemblages in aspen-dominated mixedwood forests at the Lac la Biche location, 1992–93 **A**Carabidae**B**Staphylinidae. Each symbol represents the catch from a single pitfall trap pooled over two years.

Based on data from all 12 lines of traps in two age classes of stands at Lac la Biche, carabid and staphylinid assemblages differed significantly between mature and old stands (carabids: F_1,70_ = 19.47, p = 0.013; staphylinids: F_1,70_ = 8.51, p = 0.003; Fig. 8A, B), and stand age accounted for 20.6% and 9.6% of the total variance for carabids and staphylinids, respectively. However, a strong spatial trend was found for carabids which separated into western (O2, M3) and eastern (O4, M2) stands, irrespective of age (Fig. 8A). To account for this, the RDA test of age effects on Carabidae was repeated after partitioning out differences between western and eastern stands and restricting permutations within each stand, and this resulted in a lower p-value (F_1,69_ = 30.43, p = 0.006). No carabid or staphylinid species were indicators of mature stands; however, two staphylinid species (*Mycetoporusamericanus* Erichson, 1839 [IV = 0.81, p < 0.001], *Ischnosomasplendidum* (Gravenhorst, 1806) [IV = 0.78, p < 0.001]) were significant indicators of old stands.

For carabids, the final environmental model contained seven selected variables: graminoid cover, litter biomass, diversity of tall shrubs, southern aspect > 2°, slope, *Salix* spp. cover, and forb diversity. Collectively these accounted for 38.0% of the variance in carabid species composition at Lac la Biche (Fig. 8A). Interestingly, environmental variables were mostly associated with particular stands rather than an age class. For staphylinids, nine environmental variables were selected: diversity of tall shrubs, graminoid cover, southern aspect > 5°, forb diversity, aspen cover, litter biomass, southern aspect > 2°, total shade, and white birch cover. Together they explained 17.5% of the total variation in staphylinid species composition at Lac la Biche (Fig. 8B). All selected environmental parameters had predictive value and differentiated to some extent between mature and old stands; however, some also reflected differences between the old stand 2 (graminoid cover, litter biomass and shade) and old stand 4 (*Betula* cover).

**Figure 9.**
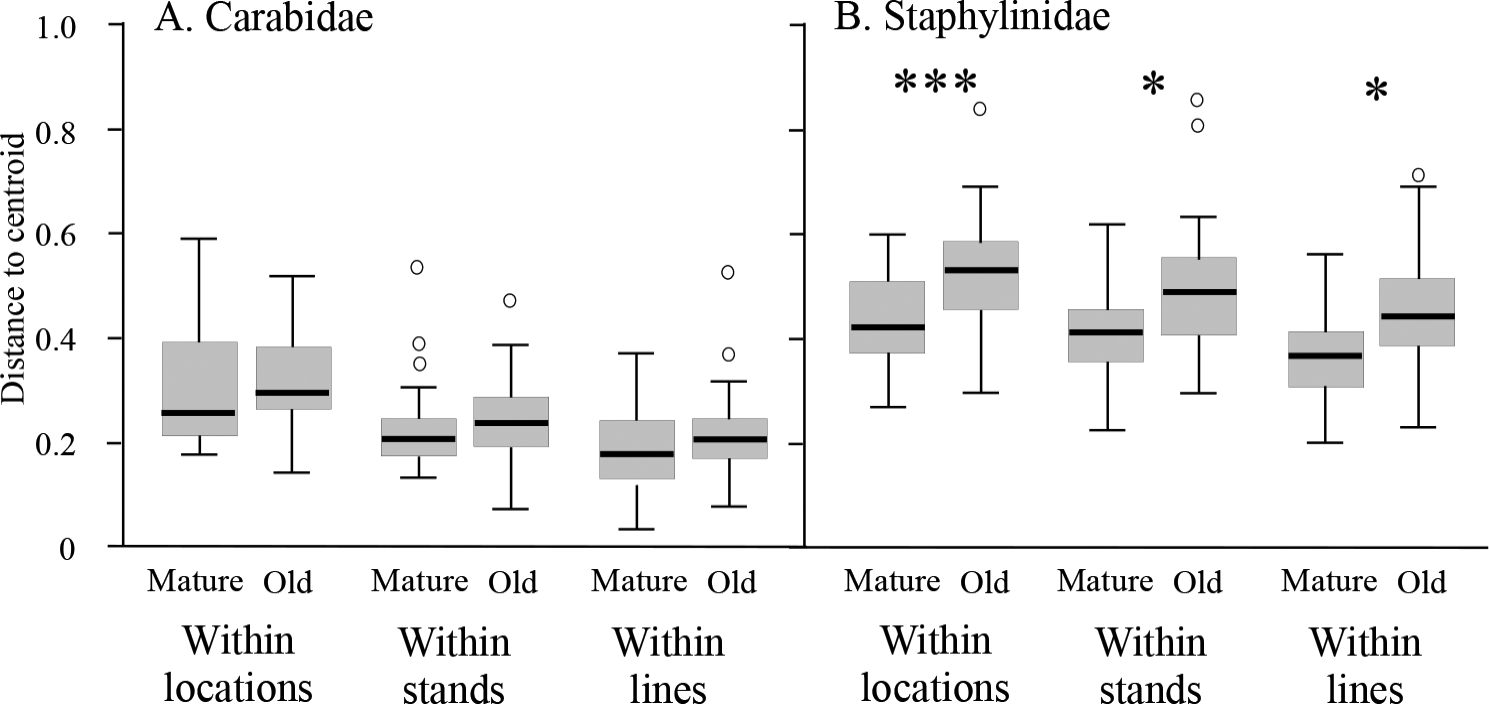
Boxplots of beta-diversity of epigaeic beetle assemblages in aspen-dominated mixedwood forests at the Lac la Biche location, 1992–93 **A**Carabidae and **B**Staphylinidae. Beta diversity was calculated within locations, within stands, and within lines for mature and old stands. Significant differences between stand age classes are indicated by asterisks above the boxplots (* p < 0.05, ** p < 0.01, *** p < 0.001)

**Figure 10.**
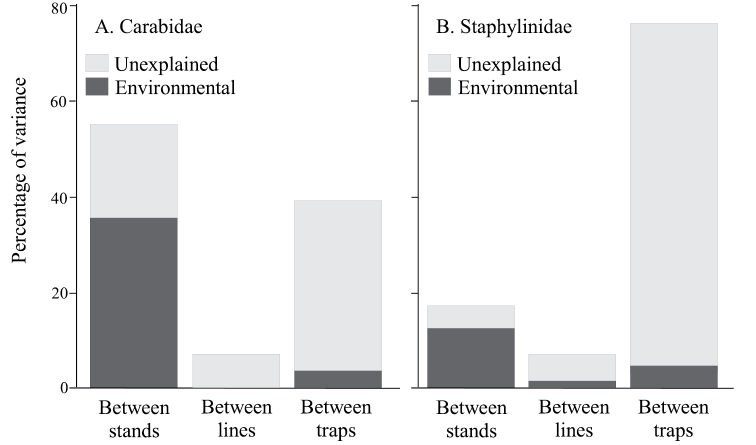
Adjusted percentage of variance of species composition in the Lac la Biche dataset explained at each spatial scale – between locations, between lines in the same location and between traps in the same line **A**Carabidae**B**Staphylinidae.

Although there was a consistent pattern of higher overall β-diversity in old than mature stands at Lac la Biche, this pattern was not significant for carabids at any spatial scale (Locations: F_1,70_ = 0.33, p = 0.763; Stands: F_1,70_ = 0.93, p = 0.378; Lines: F_1,70_ = 2.11, p = 0.163; Fig. 9A) but was significant for staphylinids at all three scales (Locations: F_1,70_ = 15.87, p < 0.001; Stands: F_1,70_ = 12.72, p = 0.021; Lines: F_1,70_ = 14.14, p = 0.023; Fig. 9B). For both taxa between-line variance was negligible (Carabids: 0.0%; Staphylinids: 6.8%; Fig. 10), providing some confidence in local assessments that apply this trapping protocol as single lines. The highest source of variance, however, differed between the two beetle families. For carabids, between-stand variance was highest at 54.7%, whereas for staphylinids between-trap variance was highest at 76.2% (Fig. 10). However, the proportion of variability explained by environmental parameters was highest for between-stand variance and this was of similar magnitude for both beetle groups (carabids: 64.7%; staphylinids: 73.3%; Fig. 10).

### Interannual variability

Overall, mean pitfall trap catches differed significantly between years for Carabidae (χ^2^ = 15.1, p < 0.001; 1992: 68.4 ± 2.9; 1993: 88.1 ± 4.7) but not for Staphylinidae (χ^2^ = 3.0, p = 0.08; 1992: 54.4 ± 3.0; 1993: 48.2 ± 2.7). Analysis of catches of both taxa showed significant interactions between years and locations (Carabidae: χ^2^ = 160.9, p < 0.001; Staphylinidae: χ^2^ = 78.0, p < 0.001). Carabid catches were significantly higher in 1992 than 1993 at Rose Creek but lower in 1992 than 1993 in both stand ages at Lac la Biche (Fig. 2A). In contrast, catches of Staphylinidae varied significantly between years only at Hinton, with catches higher in 1992 than in 1993 (Fig. 2B). There was also significant interannual variability in interactions between years and locations for either estimated species richness (Carabidae: χ^2^ = 12.5, p = 0.051; Staphylinidae: χ^2^ = 19.9, p = 0.003) or species diversity (Carabidae: χ^2^ = 17.4, p = 0.008; Staphylinidae: χ^2^ = 32.1, p < 0.001) (Fig. 3).

Epigaeic beetle composition differed significantly between years for many of the locations (Table 3). For Carabidae, assemblages were ≥ 70% similar for each location, except for old stands at Lac la Biche (56%); whereas staphylinid assemblages varied more between locations and years than did carabids, with the year-to-year similarity ranging from 51–80% (Table 3). Changing relative species weightings had mixed effects on the interpretation of the similarity of catches across years. Similarity values based on abundance for both taxa were essentially the same whether all species were included in the analysis or only species with total catch > 5 individuals (Table 3). However, use of presence-absence data that equalized species weighting, increased interannual similarity in catches of both taxa, although this was location dependent (Table 3). This suggests that less commonly collected or transient species can greatly influence measures of epigaeic beetle composition across years. In fact, several species were present in only one year at several localities, including nine carabid species at George Lake, Peace River or Rose Creek and 12–20 staphylinid species at each location. Thus, pitfall trap catches are affected each year by factors acting at the local level, and annual changes in epigaeic beetle assemblages sampled by pitfall trapping can be considerable. This highlights the importance of understanding the natural history of individual species, as interpretation of results based on species that are truly ‘rare’ may be dramatically different than on species that are simply transient or collected by chance.

**Table 3.** Interannual variation in Carabidae and Staphylinidae collected in 1992 and 1993. Comparison of faunal similarity between years is based on the standardized catch of beetles collected in 1992 and 1993 using the Bray-Curtis measure (= 1- dissimilarity value). Multivariate permutational ANOVA (999 permutations) used to test differences in similarity between trap catches across years partitioned by location. P-values = † (0.05–0.10), * (< 0.05), ** (< 0.01), *** (< 0.001).

Location	Carabidae							Staphylinidae						
	Percent similarity-catch						Number of species collected only in 1992[1993]	Percent similarity-catch						Number of species collected only in 1992[1993]
	All species		Catch > 5		Presence			All species		Catch > 5		Presence		
George Lake	0.811		0.814		0.757	*	2[7]	0.800	†	0.801	†	0.682	**	6[8]
Hinton	0.763	*	0.764	*	0.875	*	3[1]	0.594	***	0.596	***	0.762	**	19[1]
Lac la Biche-mature	0.723	***	0.723	***	0.786		3[3]	0.736	***	0.739	***	0.806	***	7[6]
Lac la Biche-old	0.557	***	0.557	***	0.857		2[3]	0.682	***	0.684	***	0.845	***	4[7]
Peace River	0.857		0.870		0.690	†	8[1]	0.507	**	0.510	**	0.818	**	6[6]
Rose Creek	0.698	**	0.699	**	0.727		6[3]	0.671	***	0.674	***	0.788		7[7]
Slave Lake	0.824		0.826		0.857		3[1]	0.623	***	0.625	***	0.824	*	3[9]

**Figure 11.**
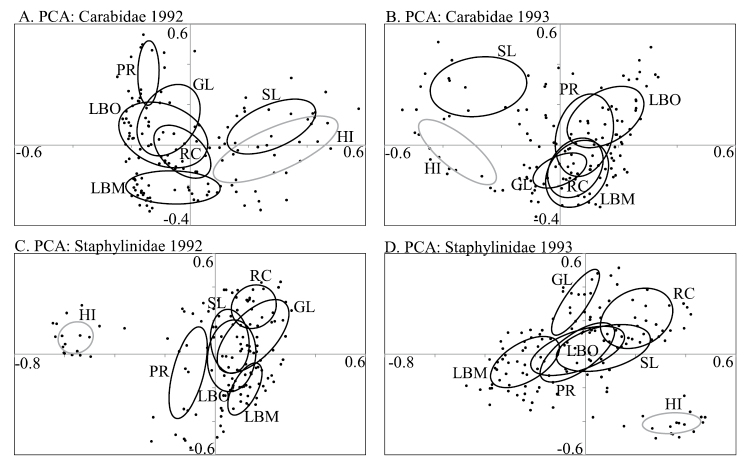
Principal components analysis of epigaeic beetle assemblages in aspen-dominated mixedwood forests in north-central Alberta, 1992–93 **A**Carabidae, 1992 **B**Carabidae, 1993 **C**Staphylinidae, 1992 **D**Staphylinidae, 1993. Each point represents the catch from a single pitfall trap. Ellipses represent the standard deviation around the centroid of points in each group.

Application of PCA ordination to carabid and staphylinid data from each year separately (Fig. 11) shows that some patterns were consistent between years but others were not. For carabids, assemblages from Hinton and Slave Lake were distinct from those at the other boreal locations in each year although these two assemblages were much closer in ordination space in 1992 than 1993 (Fig. 11A, B). Assemblages at the other five locations separated more along axis 2 in 1992 than 1993. For staphylinids, there was consistently broad separation between Hinton assemblages and those at boreal locations (Fig. 11C, D). Patterns of the boreal assemblages, however, varied between years. The assemblage at Peace River clearly differed from other boreal locations in 1992 but not in 1993, whereas the George Lake assemblage showed more separation from other locations in 1993 than 1992.

## Discussion

### Spatial variation in carabid and staphylinid assemblages

Although species richness of Staphylinidae was almost twice that of the Carabidae, both total catch and mean catch per trap was higher for the Carabidae. Mean catch, species richness, and species diversity differed among locations with no clear patterns for either taxon (Figs 2, 3). However, assemblages of both families differed significantly in composition between forest stands in the Boreal Forest and Foothills Natural Regions, and to a lesser extent among subregions of the Boreal Forest Natural Region (Fig. 4). Abundance of species varied dramatically, with fewer than three individuals collected for approximately 25% of the species. Most of these species were incidental, associated with special habitats (e.g., wetlands, coarse woody material) or were non-native species. Patterns of species dominance differed among locations. Climate variables such as the number of frost-free days, dry periods, and mean summer temperatures were identified as significantly influencing epigaeic beetle assemblages across Natural Regions; whereas vegetation, litter, shade and stand age influenced epigaeic assemblages among subregions (Fig. 5). Staphylinid β-diversity differed significantly between Natural Regions, whereas carabid β-diversity differed mainly among locations (Fig. 6). Environmental factors explained the most variation between locations and, as expected, least between traps. The amount of unexplained variation was highest between traps (Fig. 7), suggesting that unmeasured variable(s) or chance alone account for differences at the local scale.

In summary, carabid assemblages seem to respond to variation on larger spatial scales, while staphylinids respond to differences at finer spatial scales. A strong spatial trend was identified for Carabidae at Lac la Biche with distinct assemblages in eastern and western stands explaining much of the variance at the stand scale (Figs 8, 10). Staphylinids at Lac la Biche responded primarily to stand age, which was also reflected in higher β-diversity in old-growth stands (Figs 8, 9), and the amount of variation explained at the finest spatial scale, between traps (Fig. 10).

### Regional differences in beetle catch and diversity

Pitfall trap catch is strongly influenced by environmental variables such as temperature and precipitation, which affect activity (Anu et al. 2009; Jaworski and Hilszczański 2013; Pozsgai and Littlewood 2014), as well as by parameters that affect population size such as fecundity, competition, dispersal, predation, and mortality that affect abundance (Den Boer 1970; Kotze et al. 2011; Hawes et al. 2013). Thus, it is not surprising to see differences in standardized catch among localities which span a large geographic area and experience different climate regimes. Nonetheless, it is notable that the standardized catch of carabids at Peace River, the only location sampled in the Lower Boreal Highlands, was only about 27–50% of the catch at other localities. This seems to have resulted from some taxon-specific affect because the Peace River catch of carabids was also approximately half that of staphylinids, whereas at other locations carabid catches were about the same or higher than those of staphylinids. The low carabid catch cannot be attributed to an anomalous year as it was equally low in both years of sampling but staphylinid catches were not low in relation to other locations. Nonetheless, rarefaction-estimated species richness and diversity of carabids were highest in Peace River.

It seems unlikely that this pattern of low catch and high diversity of carabids is characteristic of the Lower Boreal Highlands Subregion or northern forests. Extensive subsequent sampling of carabid assemblages in aspen forests at another locality (EMEND) in the same Subregion, ca. 55–60 km to the northwest of the stands sampled in the present study, did not show unusually low catch or high species richness/diversity (Wu et al. 2020). As both stands sampled near Peace River were separated by only 2.8 km and were situated in an area including recently harvested stands, it seems that local conditions or perhaps some local landscape effects were likely responsible for low catch and high diversity of carabids. Nonetheless, such conditions did not similarly affect staphylinids at Peace River, underscoring that these two families of epigaeic beetles can express different spatial patterns of catch and diversity despite inhabiting the same general habitat.

Staphylinid diversity was highest in the three stands near Hinton in the Foothills Natural Region, likely reflecting a local mix of boreal, northern, and subalpine/montane elements (Spence et al. 1996). Furthermore, there is anecdotal evidence that mushroom abundance and diversity was higher at Hinton than at other locations during this study (Langor, pers. observ.), and given the intimate relationship between staphylinids and mushrooms (Lipkow and Betz 2005), this may have increased staphylinid catch and diversity. The higher species richness and diversity of non-aleocharine staphylinids compared to carabids at all locations is a pattern commonly reported in many forest biodiversity studies (e.g., Pohl et al. 2007, 2008) and likely reflects the high functional diversity of Staphylinidae compared to Carabidae.

The six carabid species caught most frequently in this study are generally dominant in forests across much of Canada, from Alberta eastward (Work et al. 2008). The three most dominant staphylinids in the current study were also dominant at the EMEND experimental site in boreal northwestern Alberta (Pohl et al. 2008), and were among the most abundant seven species found in coniferous forests in the Upper Foothills Natural Subregion in the Hinton area (Pohl et al. 2007). Although a core group of beetle species were found in all or most locations sampled in this study, PCA showed that the composition of the epigaeic beetle fauna differed considerably across ecoregions and locations as a result of differences in both presence and relative catch of species.

Differences between years, as documented above, can also affect interpretation of relationships among assemblages at different locations, the essence of understanding β-diversity. Of course, interannual variability, driven mainly by climactic variation, is one aspect of such differences; however, it is clear that even assemblages in more stable successional stages of forest, as we have studied here, can be expected to change progressively over time (Buddle et al. 2000, 2006; Wu et al. 2000; Belluz et al. 2021). As a reasonable compromise, we suggest that the best designs for biodiversity inventory will compare locations based on samples collected over two or more common and adjacent years, in an effort to account for interannual variability. However, analysis in this paper suggests that attempts to measure and understand differences among epigaeic beetle assemblages should rely on abundance data for reasonably common species. We found that use of presence-absence data accentuated the impact of rare, hard to sample and possibly itinerant species on assessments and comparisons. It is nonetheless important that records of rare species are kept as the fate of rare species is perhaps the most important element in understanding biodiversity loss and promoting conservation of biodiversity (Macdonald et al. 2008; Spence et al. 2008).

A significant question for conservation biologists interested in arthropod faunas is whether the structure of assemblages must be matched and maintained, or whether practices that maintain populations of all species in some combination are sufficient. Answering this question is complicated by the fact that arthropod species are differentially affected by climate change at the same time that other anthropogenic changes are also affecting natural habitats (Arribas et al. 2016; Halsch et al. 2021). Thus, we should expect that the structure of recovered assemblages will also differ from their measured pre-disturbance form, and monitoring faunal recovery in relation to assemblage structure, except in the very short term, may seem hopeless. We must take heart in Terry Erwin’s (1991) approach: inventory faunas, in so far as possible, at the species level and be on the look-out for species loss. In most boreal and temperate areas of the northern hemisphere this is possible for many arthropod taxa because of dedicated taxonomic work. We can identify faunal change by comparing new inventories with baseline records, and through comparisons with the fauna of forest preserves, better understand what is driving change. We conclude that our focus must be in understanding and conserving species.

### Regional variation in beetle assemblage structure

The most distinct and consistent spatial pattern revealed by our study is that assemblages of both carabids and staphylinids from the Lower Foothills Subregion (Hinton) differed greatly from most of those from Boreal Forest Subregions. As the single exception, carabid assemblages at Slave Lake were more similar to those at Hinton. The distinct nature of the Hinton assemblage is underscored by the high number (eleven) of indicator species for that location. There was also a major shift in dominance patterns among staphylinid species between Hinton and the boreal locations. Locations in the Boreal Forest Natural Region were dominated by *T.fumipennis*, whereas this species was largely replaced in dominance by *T.elongatus*, *T.quebecensis* Robert, 1946, *T.vergatus* Campbell, 1973, *T.thruppi* Hatch, 1957, and especially by *T.frigidus*, in the Lower Foothills (Suppl. material 1: Table S1). Most of the carabid and staphylinid species indicative of the aspen stands near Hinton are also commonly found in conifer-dominated stands in the same area (Niemelä et al. 1993; Pohl et al. 2007) suggesting that epigaeic beetle assemblages are structured more by the general foothills environment than by dominant forest type.

Nonetheless, epigaeic assemblages of some stands near Hinton were more similar to those of boreal locations. The MRT analysis for staphylinids separated all three Hinton stands from those of the boreal locations at the first split in the MRT, indicating a high level of between-stand similarity in faunal structure. However, in contrast, the MRT for carabids resulted in assemblages from the HIA and HIC stands and Slave Lake stands separating together at the first split, while the HIB stand was grouped with some boreal locations in the second split. We note that the HIB stand was closer to a highly disturbed peri-urban area near the Athabasca River and at a much lower elevation (ca. 1000 m asl) than to the other two sites that had no urban or riverine influence (1160–1190 m asl). These differences are likely to be causally associated with presence of *P.melanarius*, a synanthropic introduced species (Niemelä and Spence 1991), and relatively high catches of *Calosomafrigidum* Kirby, 1837, *Pterostichusadstrictus*, and *Pterostichuspensylvanicus* in the HIB stand. The fact that this pattern of faunal similarity was not observed for staphylinids again likely underscores differences between these families in species responses to environmental conditions.

The relationships of carabid and staphylinid faunas between Slave Lake and other locations differed as depicted in PCA ordinations. Because the two stands sampled near Slave Lake were in a transition zone between the Lower Foothills Subregion of the Foothills Natural Region and the Central Mixedwood Subregion of the Boreal Forest Natural Region (Downing and Pettapiece 2006), we expected that the epigaeic beetle fauna would also be transitional. However, this was more evident for carabid assemblages than for staphylinids. The Slave Lake carabid assemblage had a strong Lower Foothills influence and was most similar to that of Hinton. Of the five carabid species indicative of the Lower Foothills fauna at Hinton, two (*Pterostichusriparius* (Dejean, 1828), *Trechuschalybeus* Dejean, 1831) were relatively abundant at Slave Lake. In contrast, the Slave Lake staphylinid assemblage was much more similar to those of boreal locations, and none of the six staphylinid species indicative of the Lower Foothills Subregion were commonly caught at Slave Lake. The fact that assemblages of these two families responded differently to landscape characteristics suggests that they have different climatic envelopes, with some carabid species able to adapt to a wider range of conditions. It is possible that the stands at Slave Lake had microclimatic conditions tolerable for foothills carabids, but that the north-facing aspect of these stands may have been insufficient to render them suitable for the staphylinid species that we collected at Hinton.

The PCA also showed clearly that beetle assemblages varied between Natural Regions; however, Subregions within the Boreal Forest Natural Region did not consistently explain spatial differences among locations for either carabid or staphylinid assemblages. In addition, spatial patterns of association were substantially different for carabids and staphylinids. For carabids, assemblage structure at Peace River (Lower Boreal Highlands) was generally distinct from that at other locations, most of which grouped closely together irrespective of Subregion. Nonetheless, the MRT grouped assemblages from one old Lac la Biche stand (LBO2) with those from the Peace River stands. For staphylinids, however, MRT grouped assemblages at Peace River with most other boreal locations except the more southerly stands at Rose Creek and one stand at George Lake.

Separation in ordination space of the carabid assemblages from the Peace River stands from those of other boreal locations may in part be attributed to the absence of *Synuchusimpunctatus* (Say, 1823) in trap catches from Peace River. This species was present, if uncommon (12–27 specimens), at the other boreal locations. Furthermore, the capture of three specimens of *Pterostichusbrevicornis* (Kirby, 1837), a species with more northerly distribution, at Peace River contrasts with its absence in catches at other boreal locations. However, because of the very low overall carabid catch at Peace River compared to other boreal locations, small changes in relative catch of different species could result in relatively large changes in assemblage structure compared to locations with much larger catches. Thus, some of the unique structure of Peace River carabid assemblages could be simply a statistical artifact of low catch. Nonetheless, absence of *S.impunctatus* in samples from stands near Peace River seems characteristic for this Subregion as extensive sampling of mature pyrogenic aspen stands elsewhere in this Subregion between 1998 and 2013 yielded very few individuals, and frequently none, in most sample years (Wu et al. 2020).

### Environmental correlates of beetle assemblage structure

In the RDAs, climate variables explained >50% of the constrained variation for both carabids and staphylinids across regions, more than that explained by vegetation and other site characteristics. Separation of assemblages from the two Natural Regions appears to reflect the importance of climate as foothills environments are generally cooler and wetter than boreal locations. Both soil moisture content and precipitation are correlated with temperature and have been reported as a prime driver of epigaeic beetle diversity in forests by many authors (e.g., Thiele 1977; Epstein and Kulman 1990; Koivula 2002; Pearce and Venier 2006; Sroka and Finch 2006; Toivanen at el. 2014). The significant correlation of southern aspect and slope with structure of carabid and staphylinid assemblages may reflect the degree to which these factors influence local microclimatic conditions. In particular, more solar radiation reaches the ground at south facing sites in regions north of the Tropic of Cancer. This affects both food availability and plant cover in ways that influence the abundance of different arthropod groups (Tolbert 1975; Rosas-Ramos et al. 2020).

Correlation between coverage by ferns and tall shrub species such as *Salix*, *Alnus* and *Prunus* with epigaeic beetle assemblage structure suggests that both groups respond to similar environmental parameters. Several studies have shown that structure of both epigaeic beetle assemblages and plant communities (trees and understory plants) are influenced by environmental factors such as soil moisture and nutrients (Langor et al. 2006; Bergeron et al. 2011; Hammond, unpublished data). Thus, variation in conspicuous vegetation such as trees, tall shrubs, ferns, and mosses can help predict the species of epigaeic carabids and staphylinids that inhabit sites in aspen forests.

Variance partitioning showed that environmental variables explained the largest fraction of the total variance in both carabid and staphylinid assemblage structure at the largest spatial scale (i.e., between locations). Furthermore, explanatory power of these variables decreased as spatial scale decreased, irrespective of total variance at each spatial scale. Of course, this is expected because at smaller spatial scales environments become more similar; however, it may also underscore the importance of unmeasured environmental variables that are generally not included in broad forest classification schemes but may be increasingly influential in explaining variance in beetle assemblages as spatial scale decreases. Microhabitats such as amount and quality of coarse woody debris, mammal scat, mushrooms, conifer needle beds, squirrel middens, persistent pools of standing water, mineral soil exposure, etc. can influence species and catch of epigaeic beetles (Niemelä et al. 1996; Langor, unpublished data) and these vary considerably in space and most are temporally ephemeral features. The fact that a much higher amount of unexplained variance occurred at the smallest spatial scale (i.e., between traps within lines) for staphylinids than for carabids suggests that unmeasured local microhabitat variability is more important for staphylinids than for carabids.

### Regional β-diversity

The fact that β-diversity was higher for staphylinids than for carabids at every spatial scale reflects the fact that α-diversity was about twice as high for staphylinids at all spatial scales. The two exceptions to this general pattern are the within-region and within-locality β-diversity for the Foothills Natural Region, which were about the same for both beetle families (note: within-region and within-locality β-diversity is identical because there was only one location sampled (Hinton) in this Region). As our sampling covered a much larger geographic area and diversity of stand types (i.e., three Subregions) in the Boreal Forest than in the Lower Foothills, we expected that β-diversity would be higher in the Boreal Forest. This was the case for staphylinids but not carabids. We interpret this to reflect the fact that the carabid assemblage in the HIB stand at Hinton differed significantly from the other two stands, as discussed above, and this is the principal explanation for the elevated β-diversity that we observed. This also explains why within-location β-diversity for carabids was significantly higher at Hinton than at other locations that covered similar areas, while within-location β-diversity for staphylinids did not differ between regions. The similarity of within-line β-diversity among regions suggests that microhabitat variability at this scale was about the same in each region.

### Local patterns of assemblage structure and β-diversity at Lac la Biche

The expanded sampling effort in Lac la Biche stands (2–4 trap lines per stand) in comparison to other locations (1 line per stand) supported investigation of whether never-cut primeval forests (> 120 years old) harboured unique biodiversity in comparison to anticipated rotation-age forests (mature stands ca. 40–60 years; Stelfox 1995). Spence et al. (1996, 1997) and Buddle et al. (2000) have previously reported that old stands harbored unique faunal elements in several taxa. Nonetheless, this dataset is also useful for investigating β-diversity and environmental correlates of assemblage structure for carabids and staphylinids at a local level, providing a comparison to large-scale regional patterns.

Although sampling location and stand age were identified as primary drivers of epigaeic beetle assemblage structure at Lac la Biche, these factors affected the two beetle families differently. Carabid assemblages clearly separated into western and eastern groups, and within the western group there was a more distinct influence of stand age than in the eastern group. In contrast, staphylinid assemblage structure was most clearly influenced by stand age but apparently unaffected by stand location. While the influence of stand age has been previously discussed (Spence et al. 1996, 1997), the distinct separation of carabid assemblages into eastern and western groups has not. Catches of species such as *Agonumretractum*, *Platynusdecentis*, *P.adstrictus*, and *P.pensylvanicus* were much greater in the western-most stands; whereas, *Carabuschamissonis* Fischer von Waldheim, 1820, and especially *Scaphinotusmarginatus*, were caught much more frequently in the eastern-most stands (Suppl. material 1: Table S1). Eastern and western sets of sites were influenced by different landscape geography. The eastern sites were on an upland ridge near (500–4000 m) to a large water body (Touchwood Lake; 29 km^2^), whereas the western sites were at lower elevation and in close proximity to a series of drainages that empty into a smaller lake (Jackson Lake; 5.7 km^2^) and black spruce dominated wetlands. Local environmental conditions may have had a larger impact on the relative distributions of carabid species than of staphylinid species, as carabids are known to be sensitive to conditions in riparian areas (Pearce and Venier 2006; Kirichenko-Babko et al. 2020).

The RDAs indicated that differences in microclimate associated with slope, aspect and vegetation were correlated with structure of epigaeic beetle assemblages at the local and regional levels, and these factors have been reported as important for structuring epigaeic beetle communities in other studies from Alberta (Niemelä and Spence 1994; Work et al. 2004, 2008; García-Tejero et al. 2018). Carabid assemblages in the western stands, especially the old stand, were associated with higher cover of grasses, especially marsh reed grass (*Calamagrostis* spp.), and tall shrubs and greater overall amounts of litter, which are generally indicative of moister or more productive sites (Beckingham and Archibald 1996; Hart and Chen 2006). In contrast, assemblages in eastern stands were associated with greater slope and increased forb and tall shrub richness. Thus, the eastern and western stands likely represented different forest ecosites (Beckingham et al. 1996). Both carabid and staphylinid assemblages are known to vary among ecosites (Langor et al. 2006; Bergeron et al. 2011; Hammond, unpublished data). Although we detected little effect of the east-west split on staphylinid assemblages, the suite of explanatory factors for them overlaps partly with that for carabids. In fact, variance in rove beetle assemblages was explained by factors also related to ecosite. In mature stands, staphylinids were correlated with a more closed canopy, high forb richness and more southern slope, whereas the assemblage structure in old stands was strongly correlated with increased cover by graminoids and tall shrubs. This seems to underscore the probable effect of local site conditions influencing epigaeic beetle assemblages.

The apparently contradictory association of graminoids with old stands likely reflects operation of gap dynamic processes that introduce patchy openings in late successional stands (Stelfox 1995; Cumming et al. 2000). In addition, such stands generally have greater litter biomass, which is positively correlated with fungal diversity (Hammond, unpublished data), an important food source or foraging habitat for many staphylinids (Lipkow and Betz 2005; Klimaszewski et al. 2013; Stefani et al. 2016), but not thought to be as important for carabids (Lindroth 1961–1969). Forest gaps also promote immigration of open habitat species such as *Amaraimpuncticollis* (Say, 1823), *Harpalusfulvilabris* Mannerheim, 1853 and *H.reversus* Casey, 1924 which were found in the old stands, albeit in low numbers (Suppl. material 1: Table S1), thereby influencing structure of epigaeic beetle assemblages (Lange et al. 2014; Belluz et al. 2021). Also, more light penetration in open stands could differentially increase arthropod activity through temperature effects. Undoubtedly other local factors such as coarse woody material quantity and quality, soil pH, and litter cover (Work et al 2004; García-Tejero et al. 2018; Hammond et al. 2018) also affect the distribution of epigaeic beetle assemblages at a local level, but we did not measure these. Fortunately, the ecological task of explaining how epigaeic beetle assemblages vary in space is not yet finished!

Nonetheless, variance partitioning in epigaeic beetle assemblages at Lac la Biche revealed patterns similar to those evident at the regional level, suggesting that variance patterns can be generalized across spatial scales. Clearly environmental variables such as climate, plant community parameters, aspect and slope that act primarily at medium to large scales, where they are most easily measured, and they explain the majority of variance at the largest spatial scales for both families. However, variables that impact beetle populations at smaller (microhabitat) scales and are more difficult to measure have the greater influence on beetle catches at the smallest scales. This is especially true for staphylinids that seem to be more attuned to microsite differences than carabids, which are usually wide-ranging, generalist predators. Thus, both our regional and local-level data sets suggest that composition of carabid beetle assemblages is governed more by ‘coarse’ scale influences that can be considered in forest management. In contrast, staphylinid assemblages are governed more by ‘fine’ scale influences that will be perhaps more challenging to manage.

The two patterns of decreasing β-diversity with decreasing spatial scale and consistently higher β-diversity for staphylinids than carabids at each spatial scale was consistent across both the regional and local datasets. However, the β-diversity pattern with respect to stand age class was less consistent, as age class clearly affected β-diversity of staphylinids but was not apparent in our carabid data. The higher β-diversity observed in old stands compared to mature stands at all three spatial scales suggests that old stands have a higher level of microhabitat diversity that is important to many staphylinid species (Pohl et al. 2008).

## Conclusions

Although aspen-mixedwood forests are similar with respect to dominant vegetation structure across Natural Subregions in Alberta, our study has clearly shown that epigaeic beetle assemblages differ across this extensive landscape. It is also clear that these two families of beetles respond to various environmental influences at different spatial scales. This is significant for development of forest management strategies aimed to conserve biodiversity. For example, a similar study in central Europe comparing effects of spatial scale on biodiversity in managed forests concluded that results from a single region should be generalized cautiously because variation in site history and composition of epigaeic beetle communities may result in different responses to similar forest management strategies across a broad landscape (Lange et al. 2014).

β-diversity is scale dependent and can be influenced by the number and distribution of sampling sites. We have shown that considerable variation exists, even at local scales (e.g., Carabidae at Hinton), and that effective study of these patterns likely requires sampling that considers resources specific to the taxon of interest (e.g., mushrooms or dead wood for Staphylinidae). Understanding of small-scale variability is elusive, and depends to some extent on stand age and successional trajectory (García-Tejero et al. 2018). Because small scale variability may be difficult to manage according to natural patterns, unexploited forest preserves that restrict human use may be the only way to conserve natural patterns of β-diversity at small scales. This could be important in providing baselines for future assessments in support of adaptive forest management (Holling 1978; Westgate et al. 2013) because studies (Work et al. 2004, 2010) have shown that forestry operations tend to homogenize assemblages and this is an expression of loss of β-diversity.

Our study also suggests that subtle climatic variation plays a significant role in the distribution of epigaeic beetles across large mixedwood landscapes in Alberta. Therefore, systems such as the Natural Regions classification are appropriate to guide development of conservation plans for these groups. Forest classification schemes at even smaller spatial scales, such as subregions, may be helpful for carabids, but will not likely work as well for staphylinids.

Further study increasing the number of replicate stands across regions and taking account of other landscape characteristics such as aspect and slope as well as land use patterns and history may provide additional information useful for faunal conservation. However, with climate change a certainty and multiple land uses occurring across these forested landscapes, we can expect redistribution of species across forested sites and resulting changes in patterns of assemblage structure. Thus, an ongoing challenge will be to differentiate effects of disturbance and land use activities that can be managed from the effects of global change. This will require study and understanding of species in relation to baseline data such as that provided here.

## Supplementary materials

Supplementary material 1 Supplementary tables Data type species data Explanation note **Table S1.** Raw catch of Carabidae and Staphylinidae species collected from boreal aspen-dominated mixedwood forests in north-central Alberta, 1992–1993, and number of traps days per line. Species catch are summed across years of collection. Nomenclature is based on Bousquet et al. (2013). The ‘regional’ dataset contains all lines, except for Lac la Biche, where only lines M23, M32, O23 and O45 (shaded areas) were included in analysis. The ‘local’ dataset only included lines from Lac la Biche (including the shaded areas). See text for specific information regarding analyses. **Table S2.** Classification and environmental data collected for each pitfall trap in 1992. **Table S3.** Climatic data for Natural Regions, Subregions, and locations. https://binary.pensoft.net/file/556106This dataset is made available under the Open Database License (http://opendatacommons.org/licenses/odbl/1.0/). The Open Database License (ODbL) is a license agreement intended to allow users to freely share, modify, and use this Dataset while maintaining this same freedom for others, provided that the original source and author(s) are credited.H. E. James Hammond, Sergio García-Tejero, Greg R. Pohl, David W. Langor, John R. Spence
